# DNA barcodes of Antipode marine invertebrates in Bay of Biscay and Gulf of Lion ports suggest new biofouling challenges

**DOI:** 10.1038/s41598-018-34447-y

**Published:** 2018-11-01

**Authors:** L. Miralles, A. Ardura, L. Clusa, E. Garcia-Vazquez

**Affiliations:** Department of Functional Biology, University of Oviedo, C/ Julian Claveria s/n, 33006 Oviedo, Spain

## Abstract

Marine biological invasions threaten global biodiversity nowadays. In this article, we have studied fouling communities from 10 port areas of south Bay of Biscay (Atlantic Ocean) and Gulf of Lion (Mediterranean Sea). A total of 834 individuals were genetically barcoded and corresponded to 95 different species. A total of 76 native species 8 genera and 1 family were identified, 58 from the Bay of Biscay and 23 from the Gulf of Lion. Furthermore, 19 species were identified as non-indigenous or cryptogenic (18 from the Bay of Biscay and 4 from the Gulf of Lion). We found a high proportion of Antipode non-indigenous species (NIS) that represented the 19.3% of all sampled individuals and the 54.21% of NIS specimens of this study. A framework for inference of donor regions based on a phylogenetic screening of genetic sequences was proposed as a proof of concept and tested, as well as models for the relationship between NIS introductions, maritime imports and distance to NIS native range and inferred donor areas. Consistent generalized linear models (GLM) with positive association between NIS genetic diversity and distance, not with maritime growth weight imports, strongly suggest that distant NIS could pose higher invasion risk than closer species. Selection for wider tolerance ranges during the long travel –direct or stepwise, as well as environmental similarity between donor and receiving regions, may explain these results.

## Introduction

Biological invasions are thought to be the second cause of species extinction worldwide just after habitat destruction^1–3^. Prevention is the recommended approach to stop the problem, since eradication of invaders is extremely difficult, sometimes impossible and costly. To understand the particular introduction pathways and main donor areas operating in a region is therefore a priority^4,5^. However, one of the most problematic issues is to forecast the next species of concern –i.e. potential invaders - in a region. In other words, are all incoming species equally prone to settling down and thrive? The answer is, likely, no. It may depend on many factors, from intrinsic species traits to habitat conditions and biological ecosystem properties of the receptor ecosystem (e.g.^6–8^). In this context, the importance of species biogeographic origin and current distribution range should be carefully considered for appropriate management and risk prioritization^4,9,10^. The link between donor and recipient ecosystems (species introduction pathway) is determined from human activities^11^, but the distance from the source population and ecosystem similarity should also be involved when predicting the risks of invasion. The establishment of a new species would be facilitated by better survival of propagules during the travel (expected to be higher in short travels) and resemblance of environmental conditions in donor and recipient habitats (expected to be higher if they are located at the same latitude and similar ecosystem types). Vectors of introduction could be assorted^12–14^ but shipping is perhaps the main worldwide pathway of marine biological invasions because non-indigenous species (NIS) can be transported in different compartments such as inside cargo pallets, ballast tanks, sea chests, hull fouling, etc.^15,16^. Since the survival and consequently propagule pressure is expected to be negatively correlated with the distance from the donor area, for equal traffic volume ships from distant sources would pose less risk for NIS introductions than those travelling from closer regions^17^. Similarly, for equal travel time NIS species transported across longitudes (E-W) are expected to be more abundant than species transported across latitudes (N-S). However, these simple hypotheses have not been tested empirically until now.

Species’ life traits and vectors operating historically in the recipient area may serve to inferring the risk of NIS introductions therein^18^, but determining what is the real donor area of a NIS is very difficult based only on this type of information. Molecular methods can provide additional data for source population inferences only if a correct sampling of putative donor regions are made. For example the southern River Dnieper was identified as the primary source population of the round goby *Neogobius melanostomus* invasion to North America based on cytochrome b haplotypes and microsatellites analyses^19^. Some salmonid introductions in South America were traced back to North Atlantic donor stocks using microsatellites and variants of single genes^20^. The cytochrome oxidase subunit 1 gene (COI) served to identify a common source of *Gracillaria vermiculophylla* incursion in Atlantic and Pacific coasts^21^, and many other cases (e.g.^22–27^). In this study, we have used DNA Barcoding to genetically and unambiguously identify all macroinvertebrates found in port areas. That genetic information would be useful not only for identification, also to trace back the introduction pathway. The number of genetic variants (haplotypes) of a NIS might be a proxy of the number of introduction events (e.g.^28^), but could also reflect high variability in the donor population of a single or a few introductions, a stepping-stones route with long stops in previous steps^29,30^ and/or occurrence of mutations en route. Moreover, new mutations are expected to occur *in situ* on NIS populations established in a mutagenic environment –such as that of ports^31–33^.

Taking all this into account, we carried on a novel study design that follows different analytical steps: (1) to ascertain species identification of specimens collected in the field through DNA barcoding, (2) to find evidences supporting multiple introductions from distant areas as an exercise of proof of concept, (3) to compute two genetic diversity indices to be used as a proxy of the number of introduction events, (4) to test for the correlation between the NIS diversity (infra-specific) with the distance and other variables linked to the vectors and (5) to develop a model for risk estimation considering distance, latitude crosses, and maritime traffic (number of ship arrivals from a region). Although only a small subset of species could be analyzed using this full framework, we have applied it to test the hypothesis of lower propagule survival for longer travel distances and latitudinal crosses.

## Results

### NIS species, native ranges and diversity

A total of 952 specimens were sampled, 752 from Bay of Biscay and 200 from Gulf of Lion. All of them were morphologically identified and 834 individuals were successfully genetically barcoded for the cytochrome oxidase I gene, COI, and/or 16 S or 18 S rDNA, being 671 and 163 from the Bay of Biscay^34^ and Gulf of Lion^35^ respectively. The DNA sequences are available in GenBank NCBI database (https://www.ncbi.nlm.nih.gov/nucleotide/) with the accession numbers KU695268-KU695305, KU697654-KU697793, KU714729-KU714835 for COI; KU559925-KU559931 for 18 S, both from Bay of Biscay samples; and KT988316, KT988318, KT988335, KX871690-KT871694 for COI and KX129957 for 18 S from Gulf of Lion samples. Genetic identification was not possible in all individuals and 118 (12.39% of total sampled specimens) failed in DNA extraction, amplification, sequencing or BLAST identification.

Nineteen species were identified as non-indigenous or cryptogenic (Table 1). Eighteen from the eight sites in Bay of Biscay (representing the 37.6% over total sampled individuals here) and four species were found from the two sites in Gulf of Lion (representing 27.1% over the total individuals in that area). The Japanese oyster *Magallana (Crassostrea) gigas* and the Australian tubeworm *Ficopomatus enigmaticus* represented >0.5% samples in the two regions, where *Ficopomatus enigmaticus* was the most abundant NIS (10.67% over total). The Global Invasive Species Database, International Union for Conservation of Nature, Invasive Species Specialist Group (http://www.iucngisd.org/gisd/) contains six of these species in its list: the Australian/New Zealand barnacle *Austrominius (Elminius) modestus*, the sea squirt *Styela plicata*, the fouling bryozoans *Bugula neritina* and *Watersipora subatra*, and the aforementioned *Magallana gigas* and *Ficopomatus enigmaticus*. Further analyses would be focused on those species.

**Table 1:** Non Indigenous Species (NIS) and cryptogenic species found in the studied regions (BB and GL for Bay of Biscay and Gulf of Lion respectively), in percentage over the total number of individuals sampled per region (N = 671 and 200 respectively), %.

Species	Taxon	Study region	Native range	PIV	n (%)	NH	Hd	π	Natives	Cargo	Distance
*Magallana gigas*	Molluscs	BB	NW Pacific	Aquaculture	45 (6.7)	21	0.697	0.011	14.5	8.32	12.3
*Botrylloides violaceus*	Tunicates	BB	NW Pacific	Aquaculture	4 (0.60)	3	0.833	0.013	20	8.32	12.3
*Austrominius modestus*	Crustaceans	BB	New Zealand-Australia	Ships	15 (2.24)	9	0.981	0.012	9.75	14.45	13.85
*Ficopomatus enigmaticus*	Polychaetes	BB	Australia	Ships	54 (8.05)	1	0	0	10.5	14.45	9.56
*Microcosmus squamiger*	Tunicates	BB	Australia	Ships	4 (0.60)	2	0.5	0.001	17.3	14.45	10.7
*Xenostrobus securis*	Molluscs	BB	New Zealand-Australia	Ships	53 (7.90)	42	0.987	0.054	6	14.45	13.85
*Livoneca redmanii*	Crustaceans	BB	NW Atlantic	Ships	5 (0.75)	1	0	0	18	5.76	4.9
*Amphibalanus eburneus*	Crustaceans	BB	NW Atlantic	Ships	4 (0.60)	1	0	0	6	28.49	4.9
*Mytilus trossulus*	Molluscs	BB	N Atlantic, N Pacific	Ships	13 (1.94)	6	0.641	0.034	17	12.23	1.9
*Amphibalanus amphitrites*	Crustaceans	BB	Cryptogenic	Ships	5 (0.75)	1	0	0	11.5	—	—
*Amathia verticillata*	Bryozoans	BB	Cryptogenic	Ships	7 (1.04)	2	0.286	0.001	18	—	—
*Watersipora subatra*	Bryozoans	BB	Cryptogenic	Ships	11 (1.64)	5	0.855	0.031	18.67	—	—
*Bugula neritina*	Bryozoans	BB	Cryptogenic	Aquaculture	6 (0.89)	1	0	0	18	—	—
*Mytilaster minimus*	Molluscs	BB	Cryptogenic	Ships	21 (3.13)	19	0.936	0.007	16.4	—	—
*Callyspongia siphonella*	Demospongiae	BB	Red Sea	Ships	1 (0.14)	1	0	0	17	1,43	3.5
*Polydora triglanda*	Polychaetes	BB	NW Pacific	Ships	1 (0.14)	1	0	0	16	8.32	—
*Styela clava*	Tunicates	BB	NW Pacific	Ships	1 (0.14)	1	0	0	19	8.32	12.3
*Styela plicata*	Tunicates	BB	Cryptogenic	Ships	3 (0.44)	1	0	0	18	—	—
*Magallana gigas*	Molluscs	GL	NW Pacific	Aquaculture	4 (2.45))	3	0.833	0.011	21	16.69	10.5
*Ficopomatus enigmaticus*	Polychaetes	GL	Australia	Ships	35 (21.5)	1	0	0	4	4.50	9.14
*Styela plicata*	Tunicates	GL	Cryptogenic	Ships	4 (2.45)	4	1	0.042	21	—	—
*Aiptasia pulchella*	Cnidarians	GL	Cryptogenic	Ships	1 (0.7)	1	0	0	21	—	—

The presumed geographical native range is presented. Global invaders recognized by the Invasive Species Specialist Group of the International Union of Conservation of Nature are marked in bold. PIV is the possible introduction vector. NH, Hd and π are number of haplotypes, haplotype diversity and nucleotide diversity for COI sequences respectively. Natives, average number of native species found in the sampling sites in each region where the NIS was present; Cargo, percent of import tons from the NIS native range over the total regional maritime imports; Distance, distance in thousand nautical miles to the center of the putative donor region - as conservatively inferred from the geographical information of the best match reference. Species names currently recognized by WoRMS.

The number of native species varied among the sampling sites. A total of 76 native species 8 genera and 1 family were identified, 58 from the Bay of Biscay and 23 from the Gulf of Lion. Reduced native species richness (<10) was found for the sites containing *Amphibalanus eburneus*, *Austrominius modestus*, *Xenostrobus securis* in Bay of Biscay and *Ficopomatus enigmaticus* in Gulf of Lion (Table 1). The correlation between the % of a NIS and the species richness in the sampling sites was negative and highly significant (r = −0.645, P = 0.005 for 15 d.f.). In contrast, the correlations between the % of a NIS and the distance or the cargo imports from its native region were not significant (r = 0.208, P = 0.475 and r = −0.412, P = 0.143, respectively, for 12 d.f. –cryptogenic i.e. species of unknown native distribution such as some bryozoans were not considered here).

High haplotype diversity (>0.8, i.e. within-species polymorphic sequences) was obtained for *Austrominius modestus*, *Botrylloides violaceus*, *Watersipora subatra* and *Xenostrobus securis* from Bay of Biscay, and for *Magallana gigas* and *Styela plicata* from Gulf of Lion (Table 1). Nucleotide diversity was also high (>0.02) in *Mytilus trossulus*, *Watersipora subatra* and *Xenostrobus securis* from Bay of Biscay, and *Styela plicata* from Gulf of Lion. For the 10 species with high polymorphism in obtained sequences, highly significant rank correlation was found between Hd and π (r = 0.693, 8 d.f., P = 0.018), suggesting that differences in diversity among NIS were mainly due to different number of multiple introductions. However, these values and their relationships depend on the native population structure and the values of the species in their native range. Then, predictions based on these parameters should be taken with caution, if the native population structure of the species is not known.

**Figure 1:**
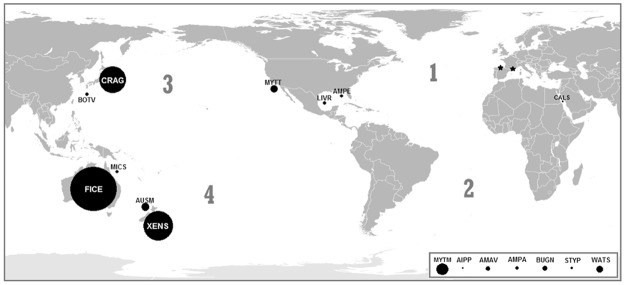
Mapamundi showing the study regions, represented with a star, and the native range centers of the most abundant NIS found therein, represented by circles of diameter proportional to the total frequency of NIS. Species acronyms are placed inside the circles. Cryptogenic NIS acronyms are placed in a box due to unknown origin. AIPP, AMAV, AMPA AMPE, BOTV, BUGN, CALS, CRAG, FICE, LIVR, MICS, MYTT, MYTM, STYP, WATS and XENS are respectively *Aiptasia pulchella*, *Amathia verticillata*, *Amphibalanus amphitrites*, *A*. *eburneus*, *Bothrylloides violaceus*, *Bugula neritina*, *Callyspongia siphonella*, *Magallana gigas*, *Ficopomatus enigmaticus*, *Livoneca redmanii*, *Microcosmus squamiger*, *Mytilus trossulus*, *Mytilaster minimus* *Styela plicata*, *Watersipora subatra* and *Xenostrobus securis*. Figure created by L. Miralles with Adobe Photoshop CS6 for Mac.

For a global spatial analysis we grouped the NIS according to their native range, with cryptogenic as one of the groups (Fig. 1, Table 2). Significant differences among groups were found for their frequency in the sampled regions, and for the average number of haplotypes (Table 2). The South Pacific group exhibited higher frequency and higher average number of haplotypes, followed by the North Pacific, North Atlantic and cryptogenic groups. Considering the two regions separately, in Gulf of Lion only Pacific species were found being South Pacific ones more abundant. In the Bay of Biscay, South Pacific NIS were also more abundant. For haplotype and nucleotide diversities the differences among groups were not significant. However, significant and positive correlation was found between mean NIS haplotype diversity and distance to the NIS native range (r = 0.643, P = 0.032 for 9 d.f.); the correlation between mean nucleotide diversity and distance to the NIS native range was not significant (r = 0.175, P = 0.607).

**Table 2:** Average proportion of NIS individuals (in percent) and sequence diversity parameters by native ocean region (SD in parentheses).

Global region	Average proportion	% BB	% GL	NH	Hd	π
North Pacific	3.098 (3.197)	7.58	2.45	9 (10.392)	0.788 (0.078)	0.012 (0.001)
South Pacific	7.251 (6.625)	18.79	21.5	11.6 (17.271)	0.614 (0.407)	0.016 (0.022)
North Atlantic	1.317 (0.751)	3.29	0	3 (2.449)	0.41 (0.496)	0.019 (0.022)
Cryptogenic	0.963 (0.426)	11.18	3.15	2 (1.732)	0.285 (0.403)	0.006 (0.014)
H (chi2):	7.395			7.97	2.616	4.015
Hc (tie corrected):	7.526			8.141	2.663	4.072
p (same):	0.023			0.017	0.264	0.131

Percentage of NIS from each region in Bay of Biscay (% BB) and Gulf of Lion (% GL). NH, Hd and π are the mean number of haplotypes, haplotype diversity and nucleotide diversity for COI sequences respectively. Statistics comparing the four regional groups based on Chi-Square tests.

### Modelling NIS diversity as a function of distance from the donor population

The Generalized Linear Models are displayed in Table 3. Considering all the exotic species that were present in our study in a proportion higher than 0.05% and the two European regions altogether (the Mediterranean region cannot be analyzed separately because *Styela plicata* and *Aptasia pulchella* are cryptogenic so only two species are left, *Magallana gigas* and *Ficopomatus enigmaticus*), we have found that local native biodiversity (measured from native species richness) was significantly correlated with NIS abundance (dependent variable *a* -as proportion of a NIS over the total number of individuals sampled from a region; G = 6.26, P = 0.012). Native biodiversity was however not significantly associated with average NIS haplotype diversity, dependent variable *b* (G = 0.041, not significant). In contrast, the distance to the native range was significantly associated with NIS haplotype diversity as a dependent variable (G = 5.214, P = 0.022). The cargo imports from a region were not significant in these models based on NIS as biological units.

**Table 3:** Generalized Linear Models for each considered variable, in the whole dataset for independent species (*a* and *b*), and in the subset of genetic-inferred donor regions (*c*, *d*).

All the species and locations					
Dependent variable *a*: NIS%	Phi	Slope	Intercept	G	P (slope = 0)
Distance	16.598	0.157 (0.245)	8.751 (1.641)	0.408	0.523
Cargo imports	1.645	−1.196 (4.356)	5.645 (1.959)	0.075	0.784
**Local native biodiversity**	**24**.**277**	**−0**.**742 (0**.**296)**	**16**.**388 (1**.**985)**	**6**.**259**	**0**.**012**
Dependent variable ***b***: NIS Hd	Phi	Slope	Intercept	G	P (slope = 0)
**Distance**	**21**.**809**	**6**.**844 (2**.**997)**	**3**.**343 (1**.**931)**	**5**.**214**	**0**.**022**
Cargo imports	1.486	−3.378 (3.696)	7.226 (2.506)	0.835	0.361
% in the region	9.798	4.08 (3.002)	0.881 (2.035)	1.847	0.174
Local native biodiversity	31.966	−1.104 (5.422)	13.87 (3.676)	0.041	0.839
**Subset with Barcode inferences**					
**Dependent variable** ***c*** **: Inferred NIS lineages** **with IS**	**Phi**	**Slope**	**Intercept**	**G**	**P (slope = 0)**
**Distance**	**13**.**563**	**0**.**328 (0**.**129)**	**1**.**379 (2**.**076)**	**6**.**428**	**0**.**011**
Cargo imports	35.128	−0.005 (0.208)	7.961 (3.340)	0.001	0.979
Latitudinal crosses	9.869	0.057 (0.110)	2.659 (1.771)	0.269	0.604
**Dependent variable** ***c*** **: Inferred NIS lineages** **without IS**	**Phi**	**Slope**	**Intercept**	**G**	**P (slope = 0)**
**Distance**	**7**.**029**	**0**.**392 (0**.**092)**	**0**.**581 (1**.**487)**	**17**.**981**	**2**.**231 × 10**^**−5**^
Cargo imports	35.071	0.021 (0.206)	7.631 (3.322)	0.010	0.919
Latitudinal crosses	8.256	0.123 (0.100)	1.845 (1.612)	1.495	0.221
Dependent variable ***d***: %NIS haplotypes					
Distance	21.648	0.170 (0.127)	3.349 (2.289)	1.787	0.181
Cargo imports	34.549	0.051 (0.161)	7.254 (2.892)	0.101	0.750
Latitudinal crosses	8.399	0.093 (0.079)	2.216 (1.426)	1.367	0.242

Distance: to the centre of the native distribution (a, b) or to the inferred donor region (*c*, *d*). Cargo imports: from the native region (*a*, *b*) or from the inferred donor region (*c*, *d*). NIS%: percentage of a NIS over the total NIS number in the study region; Hd: haplotype diversity; %NIS haplotypes: proportion of haplotypes from a donor region over the total number of NIS haplotypes, in percent. Significant variables are marked in bold.

**Figure 2:**
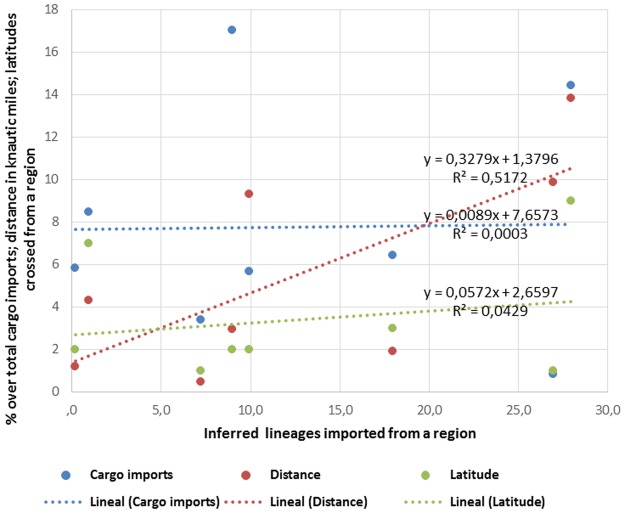
Plot representing the relationship between the number of inferred lineages from a region and different physical variables. The equation of the linear slope and the R^2^ value are displayed. Cargo imports, distance and latitude are respectively the % of cargo from an inferred donor region over the total imports, the distance in Knautic miles to the inferred donor region, and the latitudes crossed between donor and recipient region following the shorter maritime route.

The proportion of barcoding-inferred lineages native to a region (dependent variable *c)* was positively correlated with the distance to such region (G = 6.43, P = 0.011 with I.S. coefficient and G = 17.98, P = 2.231 × 10^−5^ without; Table 3). This positive correlation can be clearly seen on Fig. 2, where the lack of correlation between barcoding-inferred lineages, latitudinal crosses and cargo imports was also evidenced. The dependent variable *d*, or average NIS haplotype diversity from a donor region inferred from genetic data, did not exhibit significant regression with any of the independent variables considered.

### A proof of concept: Origin inferences from genetic data

For inferences about possible donor populations or lineages from DNA sequences, we focused on a subset of species with biogeographical references in public genetic databases and with more than 10 genetically confirmed individuals in this study, i.e. *Austrominius modestus* (n = 15), *Magallana gigas* (n = 45), *Mytilus trossulus* (n = 13), and *Xenostrobus securis* (n = 53). The inference about NIS possible donor populations from haplotype analysis (Table S1) was impossible for some species like *Styela plicata* because this cosmopolitan species does not exhibit any consistent phylogeographic pattern^36^ or *Watersipora subatra* since a recent revision of the genus considered that two species were enclosed under the name of *W*. *subtorquata*^37^ and databases were not updated in the moment of this study, thus population inferences cannot be drawn from genetic data. In the case of *Ficopomatus enigmaticus* only one confirmed geo-referenced haplotype from Australia were available in databases. For *Austrominius modestus* the geographical coverage provided by nucleotide databases was minimal (Tables S1 and S2). For the sequences of *Magallana gigas* obtained from this study, the best match sequence in BLAST for 13 haplotype was one sequence described from Portugal, within the European Atlantic Arc (the same geographic area as Spanish southwest Bay of Biscay), for other 7 haplotypes the best match were 6 referenced haplotypes from its native distribution area (Northwest Pacific) and also for one haplotype was California. In the case of *Mytilus trossulus* five of our haplotypes matched with three haplotypes corresponded to those from native distribution areas (two references from the Baltic Sea and one in Northwest Atlantic), but one was found in Australia (Tables S1 and S2). Finally, the correspondences of the studied haplotypes of *Xenostrobus securis* were one haplotype that had been reported from Galicia (northwest Spain) within the Atlantic Arc, while other five haplotypes were from Australia. However, the inferences about the donor population could not be made straightforward from the best match sequences obtained in BLAST because, as seen above, geographical coverage was incomplete for many species and phylogeographic signals were weak in some cases (Table S2). A correction coefficient (from 0 to 1) for the inference strength (IS) was employed to balance the available geographical information. Based in our data, inference strength was higher for *Mytilus trossulus* (considered as 1), and medium for *Magallana gigas* (0.5). Moderate IS (0.3) was found for *Xenostrobus securis*, weak (0.1) and very weak for *Austrominius modestus* (0.01). Applying IS as correction coefficients to the number of haplotypes from each donor population or lineage (Table S2), contributions from Pacific Ocean were slightly lower for the inferred lineages (64.8%) but higher for the total NIS haplotype variation inferred, 57.55% (versus 50.6% haplotypes). In Table S2 (bottom line) we presented also all the regional imports. No significant correlation was found between regional cargo imports –as a proxy of maritime traffic- and the relative contribution to total NIS diversity or the number of NIS lineages from a donor population (r = 0.128, P = 0.763 and r = −0.010, P = 0.981 respectively for 6 d.f.). Significant and positive correlation was found however between the number of inferred lineages from a donor population and the distance to its distribution area (r = 0.719, P = 0.04), as occurred for the individual NIS Hd (Table 1) and the distance to its native range (see above). The proportion of inferred lineages from each region was roughly similar to that obtained from the global AquaNIS database^38^ for the Atlantic Iberian coastal zone (which includes the studied region in the database): 65.4% Pacific imports were reported in AquaNIS versus 64.7 from our data, with similar distribution among large regions (Table S2, bottom half).

**Figure 3:**
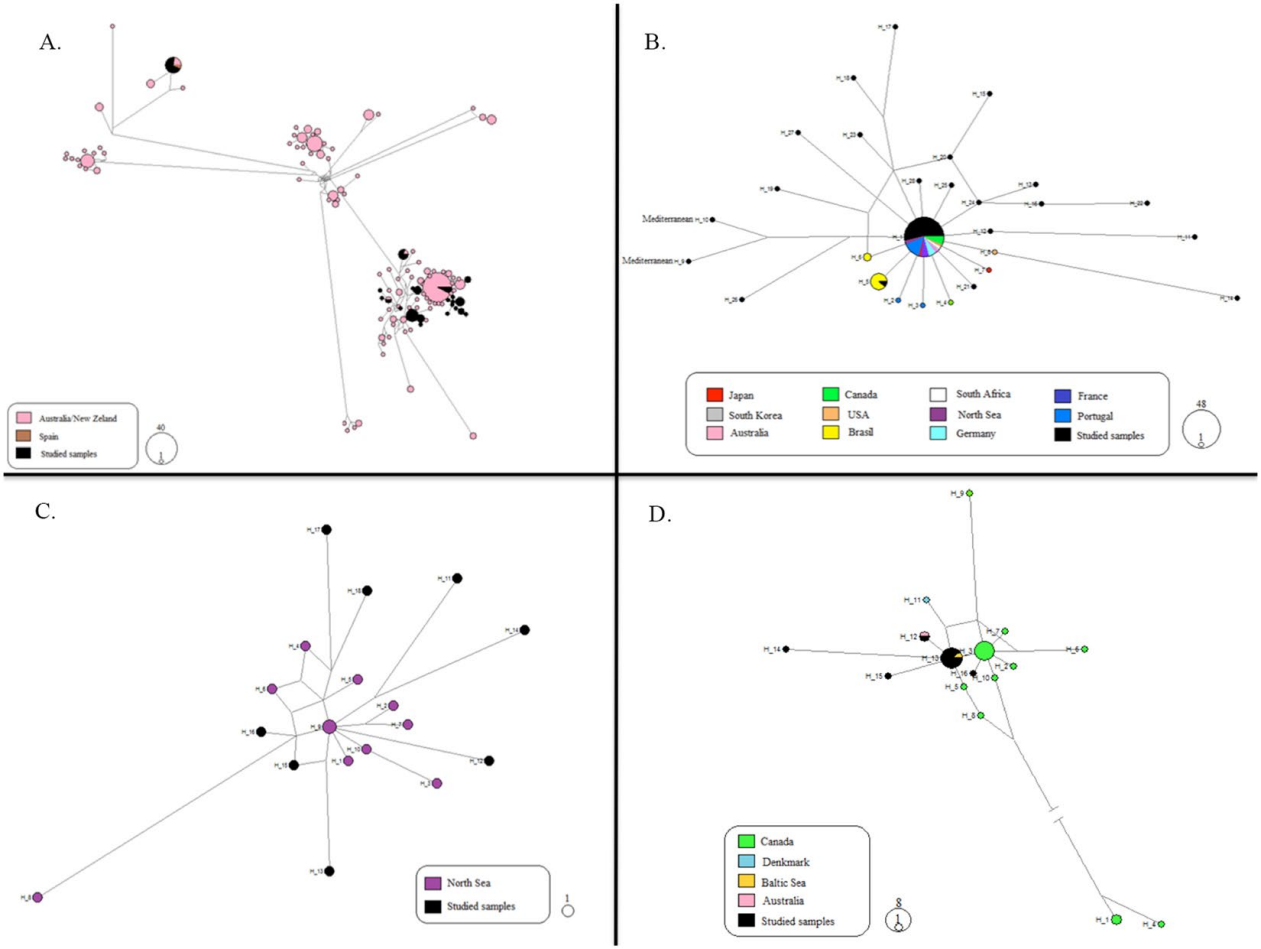
Phylogenetic Median-Joining networks of studied species with geographically referenced voucher specimens from public databases. Different regions are represented in different colors. All studied samples are in black and haplotypes from the Gulf of Lion are labeled as Mediterranean. Circle sizes are proportional to the frequency of each haplotype and branches are proportional to the number of mutations between haplotypes. A. *Xenostrobus securis* (n = 258); B. *Magallana gigas* (n = 84); C. *Austrominius modestus* (n = 19); D. *Mytilus trossulus* (n = 32).

A more refined approach for the inference of donor lineages was done from phylogenetic networks, employing the best match haplotypes obtained from BLAST and all haplotypes from public databases that had voucher specimens with geographical coverage (Fig. 3). Networks supported the rough inferences made from the model above for *Xenostrobus securis*. The haplotypes found in this study clustered principally with Australian/New Zealand haplotypes (Fig. 3A). Regarding *Magallana gigas* (Fig. 3B), the most frequent haplotype was described in many regions worldwide and geographical inferences are not possible. Also, one haplotype was identical to one reference from Brazil. The two Mediterranean haplotypes were clustered together in a separate branch suggesting a common ancestor for the two of them. The reference haplotypes of *Austrominius modestus* were all described from the North Sea where this species is introduced (Fig. 3C); the sequences found in our study were different from them and separated by different numbers of mutations. For *Mytilus trossulus* (Fig. 3D), a few haplotypes were identical to one described from Australia, while the majority clustered closer to Baltic Sea lineages.

## Discussion

In this study we have discovered positive (instead of the expected negative) correlation between number of genetic variants of a non-indigenous species and the distance to its native region, focusing on fouling species that can be transported by ships. We have found more NIS lineages native from far geographical areas than from closer ones, and this could not be explained from more intense maritime traffic from farther regions since correlation with cargo imports –a proxy of maritime traffic volume from a region – was not found. Thus, other possible explanations, such as other introduction vectors (for example aquaculture in the particular and well known case of *Magallana gigas*) or other processes (like selection during the travel, stepping-stones pathway, etc.) should be taking into account. In the models tested here, the distance to the NIS native region was the salient independent variable associated with NIS genetic diversity (as a proxy of introduction events), either taking as biological unit each NIS (dependent variable *b*), or the ensemble of NIS native to a region (dependent variable *c*). Our model was conservative employing the number of reference sequences as indicators of introduction events (dependent variable *c*). Founder effects, reflected in low genetic diversity, are expected in the recipient populations after a long-distance transfer^39,40^. Multiple invasion events of the same species^41^ could explain several haplotypes at the relatively conserved COI gene (Table 1), although indeed genetic variation of the donor population could not be ruled out. The dependent variables *b* and *d* would include all the variation, and the variable *c* would reflect the number of imports from putative donor regions. However, an interpretation based on phylogeographic inferences should take with caution, especially when nowadays the population structure of a species could reflect the influence of contemporary processes (human-driven transfers) as well as population history.

Although the two studied regions, the cold and oxygenated Cantabrian Sea in the Bay of Biscay and the warm and calm Mediterranean Sea in the Gulf of Lion, are very different ecosystems^42^, both presented NIS and invasive species in their port biota. Two of these harmful species, *Ficopomatus enigmaticus* and *Magallana gigas*, were found in both regions. This Australian tubeworm commonly adhered to vessel hulls and port structures represented the most abundant invasive species in both studied ecosystems. Our results were consistent with different lineages introduced in the two regions, where the reported introductions occurred separately in different decades^34,43^ in the Mediterranean Sea. On the other hand, the haplotypes of *Magallana gigas* found from the Mediterranean Sea in this study clustered together in a branch of the network, apart from most haplotypes found in Bay of Biscay. This could be also interpreted as different introduction events of different lineages, but gene drift processes in the population established in the Mediterranean Sea cannot be excluded as an alternative explanation. Many haplotypes of this species in Bay of Biscay, where it was introduced many decades ago and is currently farmed^34^, would suggest multiple introductions (either from aquaculture and/or shipping) in the region.

NIS from the distant Asian Pacific and Oceania were the most abundant in the two European regions studied, North Iberian and Mediterranean port areas. This can be logically explained from more *successful* introductions of those distant NIS. If confirmed from other pairs of Antipode latitudes, this effect could be called an *Antipode phenomenon of biological invasions*: species from most distant origins would adapt better in recipient ecosystems and generally exhibit higher invasiveness. One explanation to this phenomenon could be similar environmental conditions. Buckey and Catford^9^ suggested that the geographic source of introduction could determine the overall success of NIS introduction. In the present case, the species well adapted to temperate marine environment in austral latitudes could thrive in the temperate ecosystems of the northern hemisphere. The antipode region of the North Iberian coast is the New Zealand South Island, characterized by a similar photoperiod, temperatures, salinities and coastal habitats New Zealand: http://www.doc.govt.nz/nature/habitats/marine/new-zealands-marine-environment/; for the studied region: http://www.aemet.es/es/serviciosclimaticos/datosclimatologicos/valoresclimatologicos?l = 1212E&k = ast). Similar environmental conditions could explain the success of Atlantic European species in New Zealand, such as the brown trout *Salmo trutta* (e.g.^44^), the fan worm *Sabella spallanzanii* and the green crab *Carcinus maenas* (e.g.^45,46^), as well as the success of New Zealand pigmy mussel in Atlantic European coasts (e.g.^35,47^).

The biology of invasions from shipping pathway is still poorly understood and there are no clear explanations for all the biological invasions transferred by maritime traffic^48^. Our two case studies could be one of these examples regarding the ecological similarity between donor and recipient ecosystems. It can be applied clearly to the comparison of Australia/New Zealand and south Bay of Biscay, but not so much to Australia/New Zealand and the warmer Mediterranean Sea. Another explanation (probably concomitant with the previous one in the Bay of Biscay case) could be the selection for resistant individuals during the travel. Organisms transported by vessels are exposed to adverse conditions that can get extremely harsh in the case of cross-latitudinal voyage^49^. The species from a temperate region should cross the equator to arrive in the same latitude at the opposite hemisphere. Selection for resistance and in general for plasticity and flexible adaptation could be expected in such cases, but also niche change could occur following the introduction process^50^. Long travels would favor en-route selection if the travel is direct, and progressive adaptation in intermediate locations if the travel occurs through stepping-stones. This explanation could reasonably justify a high diversity of distant NIS. Following stepping-stones paths^51,52^, NIS stop first in intermediate locations and advance progressively. If different routes were employed and the steps were different, different genetics variants would be expected from each route. Stepping-stones introduction model^51,52^ would imply that organisms may travel back and forth along maritime routes, and a NIS could be imported simultaneously from different routes. The distance between donor and recipient locations -and expectedly the travel length and route as well, seem therefore to be especially important for explaining the likelihood of successful NIS introduction in recipient areas.

It may be important to note that in our models only NIS diversity, not NIS abundance (first model in Table 3) was significantly correlated with the distance to the donor region. NIS abundance may be mediated by the introduction time (older introduced NIS have reproduced in the recipient region for a longer time) and, as in the model for NIS abundance as dependent variable *a*, by native biodiversity of recipient ecosystems^34^. To date, knowing real NIS introduction time is impossible because the first NIS incursions, often occurred in early invasion stages, are almost always inadvertent and it is possible that a NIS occurs in a place for a long time before it is actually seen and inventoried^4,53^. Current exploratory methods based on environmental DNA could serve to early detection of NIS even when they are not yet visible^54,55^.

In this study, we have provided a framework for the inference of NIS geographical donor areas from genetic studies based on geographical coverage and NIS phylogeographic patterns. Such inferences were weak in many cases, thus the present study represents an exercise of proof of concept of what could be done when genetic studies and databases were complete (Table S2). On one hand, the native distribution area and even the taxonomic status of some species are uncertain in some cases. An example was *Watersipora subatra*, which complex phylogenetic and phylogeographic pattern is still under study (e.g.^27,56^). Another example was *Amathia verticillata*; its native or foreign status in Europe is also in discussion since it seems to be reported under other names from Mediterranean Sea^57^. Another possible doubt about status could affect *Mytilus trossulus*. It was native to the Pacific in the Pliocene^58^. Late Pleistocene or Holocene trans-Arctic migration(s) brought *M*. *trossulus* into the Atlantic^59–61^. Nowadays this species is widespread in the North Atlantic from the Gulf of Maine to the Arctic along the North American coast, and within the Baltic Sea^62^, as well as along the North West Pacific from the East Bering Sea to California, and is an invader in other regions^38^. For its wide distribution it might be taken as a cryptogenic; however, its native distribution is well known and widely reported, and does not include Bay of Biscay, thus we did consider it a NIS. On the other hand, determining the donor areas (either distant or close) was impossible in some cases due to limited geographical coverage of the available reference sequences. As previously suggested by Geller *et al*.^41^ and here demonstrate in different taxa, there are several conditions that challenged an accurate genetic identification of a source: (1) the existence of geographic genetic structure in the native range of the studied specie; (2) the time elapsed since the introduction; (3) inaccurate and incomplete genetic databases and (4) changes in species names and changes in taxonomic status. Improving geographical coverage, as well as available databases, is indeed a priority of barcoding projects^63^ and, from our study, would also help to manage biological invasions. Successful prevention of new invasions may be achieved only if the sources and pathways can be determined^42,64,65^. NIS genetic data with accurate regional information could be employed for researchers and managers for inferring origin of biota when databases were more complete and accurate. Even it was an exercise of proof of concept, the results obtained in this study suggest that introductions from Antipode regions should be an issue in bioinvasions management.

Last but not least, as commented above Generalized Linear Models also supported the hypothesis of biotic resistance in the two European regions studied. Local native biodiversity would confer resistance against biological invasions in a community, as proposed by other authors^55,66,67^. Prevention of new imports has demonstrated to be effective in reducing the introduction pressure^68^, but being realistic it seems impossible to achieve zero new maritime arrivals –at least in a near future. Supporting and enhancing local biota inside ports^34^, carefully controlled to prevent damage to structures and export of potentially invasive species, could be a strategy for controlling expansions of new NIS in vulnerable areas.

## Materials and Methods

### Study regions

Two European coasts very different in terms of climate and anthropogenic disturbance were considered: the Asturias coast in the south Bay of Biscay (Cantabrian Sea) and the Gulf of Lion (Mediterranean Sea). International ports within each region are Aviles and Gijon in Asturias and Marseille in Gulf of Lion. Both regions receive large cargo vessels and have smaller fishing ports and marinas. The international maritime traffic was quantified from the statistics of cargo tons of the international ports of Asturias since 1962 (website of the Spanish Government http://www.puertos.es/es-es/estadisticas/Paginas/MemoriasAnuales.aspx, accessed August 2017), and from the maritime traffic statistics in the Mediterranean Sea that can be found online at https://www.suezcanal.gov.eg/English/Navigation/Pages/NavigationStatistics.aspx and other web pages.

### Sampling and Barcoding methodology

Sampling of fouling assemblages on artificial structures followed Miralles *et al*.^34^ approach in both regions. The sampling strategy was based on the Rapid Assessment Survey (RAS) but adapted for marine invertebrates located in artificial port structures. Eight sites (Figueras, Luarca, Cudillero, Avilés, Gijón, Villaviciosa, Ribadesella and Llanes ports) with three replicates in each site were sampled in Asturias coast, and two ports (coordinates 42.67413°N-3.0134°E, 42.70201°N-3.04091°E) also with three replicates each in Gulf of Lion^35^. The three replicates were representative of wave exposure: 1 outer and 3 inner piers. For preventing biased collection of species with patchy distribution, a visual inspection prior to sampling was made to determine the phenotypically different organisms (presumably different species) present in the sampling site. To standardize the sampling effort, the surface sampled from each site within each port was approximately 200 m^2^. Roughly 1% of the animals visually detected attached on that surface were collected at random. To equalize the sampling effort, each site was sampled for 30 min. and a maximum of 100 individuals were taken per port. The number of individuals picked of each morphotype was approximately proportional to the abundance of such morphotype. Individuals were collected from artificial structures by scratching the surfaces with a small trowel and immediately putting in a plastic bag with seawater. All individuals were taken to the laboratory for morphological and genetic species identification. Individuals were previously divided based on different morphological phenotypes. Then, a piece of tissue from each individual was ethanol-preserved for genetic analysis. Voucher specimens were stored in ethanol in the Laboratory of Natural Resources of the University of Oviedo (Asturias and Gulf of Lion samples).

DNA was immediately extracted from the ethanol-preserved individuals and barcoding regions (cytochrome oxidase I gene, COI, for all species; and 18 S or 16 S rRNA genes for taxa when COI amplification failed) were PCR-amplified and sequenced as described in Miralles *et al*.^34^. DNA sequences were edited with BioEdit v7.2.5^69^ and compared online with reference public databases using nBLAST in NCBI (www.ncbi.nlm.nih.gov/) and Bold Systems (www.boldsystems.org/). Haplotypes were determined with DnaSP software v5.10^70^. For further analyses, only specimens with both types of identification (genetic and morphological) were included in this study.

The Invasive Species Specialist Group (ISSG) global database (http://www.issg.org/database/welcome/) of the International Union for Conservation of Nature (IUCN) and the AquaNIS (Information system on aquatic non-indigenous and cryptogenic species database) were the reference to check the introduced and invasion status of each identified non-indigenous species, when available information made it possible. The native range of those NIS was taken from the Global Invasive Species Database, AquaNIS, DAISIE and NEMESIS databases (accessed in September 2018). The taxonomic nomenclature of all identified species was verified against the World Register of Marine Species – WORMS^71^.

### Modelling and statistical analysis

The number of lineages imported from a region, as a proxy of the number of introductions, was estimated using the number of best match reference sequences retrieved from public databases (identity >99%, E-value of zero as explained above). The weighted haplotypes represented the *in situ* (in the introduced area) variation of NIS from a region. These two parameters were analyzed as percentages over the total number found in the case studies, for standardized statistics. Distance to putative native range was calculated considering the most important route, which is the most direct in many cases. In case of disjoint distribution, the most conservative route was selected.

Pairwise correlations were first performed with PAST software^72^ version 3.11 for Mac OSX 10.8 and later, to identify possible internal correlations between variables. One variable from each pair with r > 0.8 was excluded from further Generalized Linear Model (GLM) analysis. GLM for single explanatory variables assumed a normal distribution, with different dependent and independent variables, in pairs. Normal distribution and log link functions were used to fit the logarithmic function. Autocorrelations were tested from Durbin-Watson statistic based on residuals from OLS regression, and homoskedasticity was tested from Breusch-Pagan tests.

Dependent variables were: *a*, proportion of a NIS over the total number of samples from a sampling point; *b*, haplotype diversity or Hd; *c*, inferred lineages from a donor region, estimated with IS from the number of NIS as explained above and also without IS; *d*, %NIS haplotypes as the proportion of haplotypes from a donor region over the total number of NIS haplotypes.

Haplotype and nucleotide diversity (Hd and π respectively) were estimated using DNAsp v.5.10^70^. Haplotype diversity is a measure of the genetic diversity and represents the uniqueness of a particular haplotype in a population, while nucleotide diversity measures the average proportion of nucleotide differences between sequences within a sample.

### Inferences of donor populations: a proof of concept

A phylogeographic approach was followed based on Median-Joining networks of each NIS with more than 10 barcoded individuals in our study and available referenced information in databases. The haplotypes of this study, their best match obtained in nBLAST and all available sequences associated with voucher specimens and geographical reference from public genetic databases were selected to build haplotypic networks using Network v.5 (www.fluxus-engineering.com) Default settings were computed to reconstruct all possible, shortest, least complex, phylogenetic trees (maximum parsimony).

Different trajectories could be involved in a NIS introduction (e.g. shipping, recreational boats, secondary spread from neighbor areas, etc.), thus it is not possible to make straightforward geographical inferences of donor regions. Instead, the population or lineage source was inferred from DNA sequences as long as: 1) The species exhibited differences between regions for the DNA marker/s employed, that is, a well differentiated phylogeographic pattern for assigning DNA variants to a region; 2) There was acceptable coverage of the distribution area of the species for genetic data; 3) Genetic and geographic data are associated and available in public repositories, that is, geographically referenced sequences with referenced voucher species specimens. Only best matches, i.e. identical or extremely similar haplotypes (99–100% identity, maximum scores for the DNA fragment employed and species, E-value of zero) were used for geographic inferences. If more than one reference was retrieved with identical score, conservative inference was done allocating best match with the geographically closer region. Distances were calculated in thousand nautical miles to the center of the putative donor region - as conservatively inferred from the geographical information of the best match reference. Furthermore, we had considered the most important maritime transport route, which was the most direct in many cases.

For inferences about the introduction history of a NIS we have considered separately: the haplotypes previously reported from other geographic areas, which is the most direct source of geographical inference, and the covariation of nucleotide (π) and haplotype (Hd) diversity. In general, high π and Hd would reflect multiple introductions from different donor populations, while low π and high Hd would reflect *in situ* mutations or expansive donor populations but less likely multiple introductions from genetically different donors, and high π together with low Hd would be consistent with only a few introductions of genetically very distinct lineages. Each NIS could have a different genetic history and phylogeographic structure of the studied species should be well examined in the native range.

The inference strength (IS) was the likelihood of the inferred population origin, and was based as explained above from population differentiation of a species (i.e. its phylogeographic signature), and coverage of the distribution area with sequences in the references databases. This coefficient was weighted from 0 to 1 according to available information. It was estimated as: 0 for species with known absence of phylogeographic patterns; 0.01 for species with minimal geographical coverage of DNA sequences and unknown phylogeographic pattern; 0.1 for species with moderate geographical coverage of sequences and shallow phylogeographic signature (shallow regional genetic differentiation); 0.3 for medium geographical coverage and moderate phylogeographic signature; 0.5 for species with good coverage and shallow phylogeographical signature; 1 for the best possible inference, species with strong phylogeographic signature and at least medium coverage. Inferences were made multiplying the number of individuals of a species by its corresponding IS. Analyses were done with and without IS coefficient to test the robustness of the analysis.

## Electronic supplementary material

Supplementary tables S1, S2, S3
