# Supplementary material for: DNA barcodes of Antipode marine invertebrates in Bay of Biscay and Gulf of Lion ports suggest new biofouling challenges
Source: Sci Rep. 2018 Nov 1;8:16214. doi: 10.1038/s41598-018-34447-y (PMC6212436; doi:10.1038/s41598-018-34447-y)
Supplement: Supplementary file 1 — Supplementary tables S1, S2, S3 [file 41598_2018_34447_MOESM1_ESM.docx]

**DNA Barcodes of Antipode marine invertebrates in Bay of Biscay and Gulf of Lion ports suggest new biofouling challenges.**

L. Miralles^1*^, A. Ardura^2^, L. Clusa^1^, E. Garcia-Vazquez^1^.

Supplementary Materials:

**Table S1.** Haplotypes found in this study and BLAST results. The reference of the best match is given, as well as its origin as stated in the sequence description. The closest voucher match referred to data with voucher specimens and geographical reference. The BLAST results include the percentage of nucleotide identity between our Barcode and its best match, the query coverage and the score.

|  |  | Closest voucher match | | BLAST results | | |  |
| --- | --- | --- | --- | --- | --- | --- | --- |
| Species | Haplotype | Reference | Origin | Identity | Query cover | Score | Comments |
| *Amathia verticillata* | Av01 | JF490060 | Brazil | 99% | 100% | 863 |  |
|  | Av03 | JF490060 | Brazil | 99% | 100% | 857 |  |
| *Austrominius modestus* | Am73R | KT208763.1 | North Sea | 99% | 99% | 806 |  |
|  | Am74R | KT208763.1 | North Sea | 99% | 100% | 813 |  |
|  | AmA02 | KT208763.1 | North Sea | 100% | 100% | 835 |  |
|  | AmR1J3 | KT208763.1 | North Sea | 99% | 100% | 813 |  |
|  | AmR1J4 | KT208763.1 | North Sea | 99% | 100% | 802 |  |
|  | AmR1J5 | KT208763.1 | North Sea | 99% | 100% | 824 |  |
|  | AmR1J6 | KT208763.1 | North Sea | 99% | 100% | 824 |  |
|  | AmR1K4 | KT209245.1 | North Sea | 99% | 100% | 813 |  |
|  | AmL3H1 | KT209245.1 | North Sea | 99% | 100% | 819 |  |
| *Magallana gigas* | Cg72 | AB736406 | Japan | 99% | 100% | 464 |  |
|  | Cg78 | KP067893 | Korea | 99% | 100% | 464 |  |
|  | Cg03 | KP067896.1 | Korea | 100% | 100% | 481 |  |
|  | Cg76 | KP099022 | China | 99% | 100% | 464 |  |
|  | Cg83 | KP099022.1 | China | 100% | 100% | 481 |  |
|  | CgLlA2 | KP099041 | China | 100% | 100% | 481 |  |
|  | CgR1F6 | KP099044 | China | 100% | 100% | 481 |  |
|  | Cg67 | KT317439 | California | 100% | 94% | 455 |  |
|  | Cg07 | KT932100.1 | Portugal | 100% | 88% | 425 |  |
|  | Cg15 | KT932100.1 | Portugal | 98% | 99% | 457 |  |
|  | CgRib1F | KT932100.1 | Portugal | 99% | 100% | 475 |  |
|  | Cg35 | KT932100.1 | Portugal | 99% | 100% | 470 |  |
|  | Cg75 | KT932100.1 | Portugal | 99% | 100% | 470 |  |
|  | Cg77 | KT932100.1 | Portugal | 99% | 100% | 462 |  |
|  | Cg82 | KT932100.1 | Portugal | 99% | 100% | 470 |  |
|  | Cg84 | KT932100.1 | Portugal | 98% | 100% | 459 |  |
|  | Cg87 | KT932100.1 | Portugal | 99% | 100% | 470 |  |
|  | Cg89 | KT932100.1 | Portugal | 99% | 100% | 475 |  |
|  | CgR1F2 | KT932100.1 | Portugal | 98% | 100% | 459 |  |
|  | CgR1F10 | KT932100.1 | Portugal | 99% | 100% | 464 |  |
|  | CgR2D | KT932100.1 | Portugal | 99% | 100% | 475 |  |
| *Ficopomatus enigmaticus* | **Fe01*** | DQ317115 | Australia | 100% | 100% | 767 | ***: 18S rRNA gene** |
|  | **FeM** | DQ317115 | Australia | 100% | 83% | 654 | ***: 18S rRNA gene** |
| *Mytilus trossulus* | MtF3T2 | DQ198225.2 | Baltic Sea | 99% | 100% | 961 |  |
|  | MtG3C7 | HQ864843 | Australia | 100% | 100% | 691 | Mytilus edulis complex |
|  | MtL3A3 | KF644025 | Canada East Coast | 99% | 100% | 686 |  |
|  | MtV3K4 | KM192134 | Baltic Sea | 99% | 100% | 664 | Masculinized (male haplotype) |
|  | MtC30 | KM192134 | Baltic Sea | 100% | 100% | 691 | Masculinized (male haplotype) |
|  | MtG3C4 | KM192134 | Baltic Sea | 99% | 98% | 1061 |  |
| *Styela plicata* |  |  |  |  |  |  |  |
|  | B5-89 | HQ916426 | Worldwide distribution | 99% | 100% | 1031 |  |
|  |  | FJ528632 | Worldwide distribution | 99% | 100% | 1031 |  |
|  | B1-20 | HQ916426 | Worldwide distribution | 97% | 100% | 948 |  |
|  |  | FJ528632 | Worldwide distribution | 97% | 100% | 948 |  |
|  | B5-Asc1 | HQ916426 | Worldwide distribution | 97% | 100% | 942 |  |
|  |  | FJ528632 | Worldwide distribution | 97% | 100% | 942 |  |
|  | B5-Asc2 | HQ916426 | Worldwide distribution | 97% | 100% | 843 |  |
|  |  | FJ528632 | Worldwide distribution | 97% | 100% | 843 |  |
| *Watersipora subtorquata* | WsG3C13 | AY647167 | California | 99% | 97% | 638 |  |
|  | WsF2E1 | DQ417453 | California | 99% | 99% | 654 |  |
|  | WsF2E2 | DQ417453 | California | 99% | 97% | 632 |  |
|  | WsF2J | DQ417453 | California | 99% | 97% | 632 |  |
|  | WsC3J | JF950413 | New Zealand | 98% | 96% | 604 |  |
| *Xenostrobus securis* | Xs07 | KC509694 | Australia | 99% | 98% | 985 |  |
|  | Xs08 | KC509694 | Australia | 99% | 98% | 979 |  |
|  | Xs09 | KC509694 | Australia | 99% | 98% | 990 |  |
|  | Xs25 | KC509694 | Australia | 99% | 97% | 987 |  |
|  | Xs26 | KC509694 | Australia | 99% | 97% | 987 |  |
|  | Xs27 | KC509694 | Australia | 99% | 97% | 992 |  |
|  | Xs03 | KC509698 | Australia | 100% | 98% | 1002 |  |
|  | Xs04 | KC509698 | Australia | 99% | 97% | 987 |  |
|  | Xs05 | KC509698 | Australia | 99% | 98% | 985 |  |
|  | Xs06 | KC509698 | Australia | 99% | 98% | 985 |  |
|  | Xs10 | KC509698 | Australia | 99% | 98% | 990 |  |
|  | Xs20 | KC509698 | Australia | 100% | 97% | 998 |  |
|  | Xs21 | KC509698 | Australia | 99% | 97% | 970 |  |
|  | Xs22 | KC509698 | Australia | 98% | 98% | 941 |  |
|  | Xs23 | KC509698 | Australia | 99% | 98% | 990 |  |
|  | Xs24 | KC509698 | Australia | 99% | 97% | 992 |  |
|  | Xs38 | KC509698 | Australia | 99% | 97% | 992 |  |
|  | Xs39 | KC509698 | Australia | 99% | 98% | 996 |  |
|  | Xs40 | KC509698 | Australia | 99% | 98% | 990 |  |
|  | Xs41 | KC509698 | Australia | 99% | 98% | 996 |  |
|  | Xs32 | KC509718 | Australia | 100% | 98% | 1002 |  |
|  | Xs33 | KC509718 | Australia | 100% | 97% | 998 |  |
|  | Xs02 | KC509720 | Australia | 98% | 98% | 946 |  |
|  | Xs11 | KC509720 | Australia | 98% | 98% | 941 |  |
|  | Xs12 | KC509720 | Australia | 98% | 98% | 935 |  |
|  | Xs13 | KC509720 | Australia | 98% | 97% | 926 |  |
|  | Xs14 | KC509720 | Australia | 98% | 97% | 926 |  |
|  | Xs15 | KC509720 | Australia | 98% | 97% | 931 |  |
|  | Xs16 | KC509720 | Australia | 98% | 97% | 931 |  |
|  | Xs28 | KC509720 | Australia | 98% | 97% | 942 |  |
|  | Xs29 | KC509720 | Australia | 98% | 97% | 931 |  |
|  | Xs17 | KC509725 | Australia | 99% | 97% | 981 |  |
|  | Xs18 | KC509725 | Australia | 99% | 98% | 968 |  |
|  | Xs30 | KC509725 | Australia | 99% | 98% | 990 |  |
|  | Xs31 | KC509725 | Australia | 99% | 98% | 985 |  |
|  | Xs42 | KC509725 | Australia | 99% | 98% | 979 |  |
|  | Xs01 | KC509740 | Australia | 100% | 98% | 1002 |  |
|  | Xs19 | KC509740 | Australia | 99% | 98% | 990 |  |
|  | Xs34 | KC509740 | Australia | 99% | 97% | 992 |  |
|  | Xs35 | KC509740 | Australia | 100% | 98% | 1002 | 100% with FJ949108 (Galicia) with 86% coverage |
|  | Xs36 | KC509740 | Australia | 99% | 97% | 992 | 100% with FJ949108 (Galicia) with 86% coverage |
|  | Xs37 | KC509740 | Australia | 99% | 97% | 987 | 100% with FJ949108 (Galicia) with 86% coverage |

**Table S2:** Geographical inferences for the NIS found in this study. N, sample size. Regional difference: level of regional genetic differentiation previously reported. Coverage: geographical coverage from the number of marine regions with referenced COI gene (or 18S rDNA for *Ficopomatus enigmaticus*) sequences in GenBank (http://www.ncbi.nlm.nih.gov/genbank/) in the moment of this study (June 2016). Inference strength (IS), likelihood of the inferred origin from phylogeographic structure and coverage. Haplotypes found *in situ* with best match to a regionally assigned GenBank reference // number of such reference haplotypes. Inferred imported lineages from a region, as IS x total number of reference haplotypes from that region, for each species. Contribution from each donor region to total NIS diversity, in % of haplotypes. Number (percentage) of marine NIS introductions reported from each region in Iberian coastal zone, taken from international databases. For GenBank search old and current synonymous species names were considered. “a” to “g” are Nascimento et al. (28), Moehler et al. (73), Zbawicka et al. (74), Pineda et al. (36), Mackie et al. (56), Pascual et al. (75), AquaNIS (38). *: Parsimonious assignation to the closer site in case of identical haplotypes occurring in different regions (see table S1).

|  |  |  |  |  | In situ haplotypes // reference haplotypes from a region | | | | | | | |
| --- | --- | --- | --- | --- | --- | --- | --- | --- | --- | --- | --- | --- |
| Species | N | Phylogeographic structure | Coverage | IS | Atlantic Arc | Baltic Sea | North Sea | NW Atlantic | SW Atlantic | NW Pacific | NE Pacific | Australia-NZ |
| *Amathia verticillata* | 7 | Shallow a | Moderate | Weak (0.1) |  |  |  |  | 2 // 1 |  |  |  |
| ***Austrominius modestus*** | 15 | Unknown | Minimal | Unlikely (0.01) |  |  | 9 // 2 |  |  |  |  |  |
| ***Magallana gigas*** | 45 | Shallow b | Good | Medium (0.5) | 13 // 1 |  |  |  |  | 7 // 6 | 1 // 1 |  |
| *Ficopomatus enigmaticus* | 54 | Unknown | Minimal | Unlikely (0.01) |  |  |  |  |  |  |  | 2 // 1 |
| ***Mytilus trossulus*** | 13 | Marked c | Medium | Strong (1) |  | 4 // 2 |  | 1 // 1 |  |  |  | 1 // 1 |
| *Styela plicata* | 4 | Inexistent d | Good | Impossible | - | - | - | - | - | - | - | - |
| *Watersipora subatra* | 11 | In study | Medium | Impossible | - | - | - | - | - | - | - | - |
| ***Xenostrobus securis*** | 53 | Moderate f | Medium | Moderate (0.3) | 3 // 1 * |  |  |  |  |  |  | 39 // 6 |
|  |  |  |  | Total | 16 // 2 | 4 // 2 | 9 // 2 | 1 // 1 | 2 // 1 | 7 // 6 | 1 // 1 | 42 // 8 |
|  |  | Inferred imported lineages (% over total) | | | 0.8 (7.2) | 2 (17.9) | 0.02 (0.2) | 1 (8.98) | 0.1 (0.9) | 3 (26.9) | 0.5 (9.9) | 2.81 (27.9) |
|  |  |  |  |  |  |  |  |  |  |  |  |  |
|  |  | Inferred contribution to total NIS variation | | | 24.76 | 13.38 | 0.3 | 3.35 | 0.67 | 11.71 | 5.69 | 40.15 |
|  |  | Cargo (% over total imports) | | | 3.41 | 6.42 | 5.81 | 17.05 | 9.5 | 0.83 | 5.68 | 14.45 |
| Number (%) of NIS introductions in Iberian coastal waters (g) | | | | | 7 (12.7) | | | 6 (10.91) | 6 (10.91) | 14 (25.45) | 6 (10.91) | 16 (29.1) |

**Table S3.** All native species found in each sampling area and barcoding method employed to identify them

| Taxa | *Species* | Sampling area | n | Barcoding |
| --- | --- | --- | --- | --- |
| Mollusca, Neoloricata, Acanthochitonidae | *Acanthochitona crinita* | BB | 1 | COI |
| Bryozoa, Gymnolaemata, Vesiculariidae | *Amathia imbricata* | BB | 1 | 18S |
| Arthropoda, Malacostraca, Ampithoidae | *Ampithoe rubricata* | BB | 1 | COI |
| Cnidaria, Anthozoa, Actiniidae | *Anemonia sp.* | BB | 1 | COI |
| Chordata, Ascidiacea, Ascidiidae | *Ascidiella aspersa* | BB | 3 | COI |
| Chordata, Ascidiacea, Styelidae | *Botryllus schlosseri* | BB | 4 | COI |
| Arthropoda, Cirripedia, Chthamalidae | *Chthamalus montagui* | BB | 15 | COI |
| Arthropoda, Cirripedia, Chthamalidae | *Chthamalus stellatus* | BB | 6 | COI |
| Mollusca, Gastropoda, Rissoidae | *Cingula trifasciata* | BB | 1 | COI |
| Arthropoda, Malacostraca, Diogenidae | *Clibanarius erythropus* | BB | 1 | COI |
| Bryozoa, Gymnolaemata, Cryptosulidae | *Cryptosula pallasiana* | BB | 1 | COI |
| Nemertea, Enopla, Emplectonematidae | *Emplectonema gracile* | BB | 3 | COI |
| Arthropoda, Malacostraca, Hyalidae | *Fam. Hyalidae* | BB | 7 | COI |
| Mollusca, Gastropoda, Chromodorididae | *Felimare villafranca* | BB | 2 | COI |
| Mollusca, Gastropoda, Trochidae | *Gibbula umbilicalis* | BB | 44 | COI |
| Annelida, Polychaeta, Nereididae | *Hediste diversicolor* | BB | 1 | COI |
| Porifera, Demospongia, Halichondriidae | *Hymeniacidon perlevis* | BB | 2 | COI |
| Porifera, Demospongia, Halichondriidae | *Hymeniacidon sp.* | BB | 4 | COI |
| Mollusca, Gastropoda, Discodorididae | *Jorunna tomentosa* | BB | 1 | COI |
| Annelida, Polychaeta, Spionidae | *Laonice cirrata* | BB | 1 | COI |
| Mollusca, Bivalvia, Lasaeidae | *Lasaea adansoni* | BB | 2 | COI |
| Annelida, Polychaeta, Eunicidae | *Leodice harassii* | BB | 1 | COI |
| Mollusca, Polyplacophora, Lepidochitonidae | *Lepidochitona cinerea* | BB | 1 | COI |
| Mollusca, Gastropoda, Littorinidae | *Littorina saxatilis* | BB | 4 | COI |
| Mollusca, Gastropoda, Littorinidae | *Littorina sp.* | BB | 1 | COI |
| Mollusca, Gastropoda, Littorinidae | *Melarhaphe neritoides* | BB | 2 | COI |
| Arthropoda, Malacostraca, Corophiidae | *Monocorophium insidiosum* | BB | 6 | COI |
| Chordata, Ascidiacea, Polyclinidae | *Morchellium argus* | BB | 1 | COI |
| Mollusca, Bivalvia, Mytilidae | *Mytilaster minimus* | BB | 21 | COI |
| Mollusca, Bivalvia, Mytilidae | *Mytilus edulis* | BB | 3 | COI |
| Mollusca, Bivalvia, Mytilidae | *Mytilus galloprovincialis* | BB | 46 | COI |
| Mollusca, Bivalvia, Mytilidae | *Mytilus sp.* | BB | 128 | COI |
| Mollusca, Gastropoda, Nassariidae | *Nassarius incrassatus* | BB | 2 | COI |
| Annelida, Polychaeta, Nereididae | *Neanthes fucata* | BB | 2 | COI |
| Annelida, Polychaeta, Nereididae | *Nereis falsa* | BB | 2 | COI |
| Cnidaria, Hydrozoa, Campanulariidae | *Obelia geniculata* | BB | 1 | COI |
| Echinodermata, Ophiouroidea, Ophiotrichidae | *Ophiothrix sp.* | BB | 1 | COI |
| Mollusca, Bivalvia, Ostreidae | *Ostrea edulis* | BB | 3 | COI |
| Arthropoda, Malacostraca, Palaemonidae | *Palaemon elegans* | BB | 3 | COI |
| Arthropoda, Malacostraca, Palaemonidae | *Palaemon serratus* | BB | 1 | COI |
| Echinodermata, Echinoidea, Parechinidae | *Paracentrotus lividus* | BB | 1 | COI |
| Mollusca, Gastropoda, Patellidae | *Patella aspera* | BB | 5 | COI |
| Mollusca, Gastropoda, Patellidae | *Patella depressa* | BB | 11 | COI |
| Mollusca, Gastropoda, Patellidae | *Patella vulgata* | BB | 32 | COI |
| Arthropoda, Cirripedia, Balanidae | *Perforatus perforatus* | BB | 3 | COI |
| Mollusca, Gastropoda, Hydrobiidae | *Peringia ulvae* | BB | 1 | COI |
| Mollusca, Gastropoda, Trochidae | *Phorcus articulatus* | BB | 2 | COI |
| Mollusca, Gastropoda, Trochidae | *Phorcus lineatus* | BB | 16 | COI |
| Arthropoda, Malacostraca, Pilumnidae | *Pilumnus hirtellus* | BB | 1 | COI |
| Annelida, Polychaeta, Nereididae | *Platynereis dumerilii* | BB | 24 | 18S |
| Echinodermata, Echinoidea, Parechinidae | *Psammechinus miliaris* | BB | 2 | COI |
| Nemertea, Anopla, Lineidae | *Riseriellus occultus* | BB | 2 | COI |
| Arthropoda, Malacostraca, Sphaeromatidae | *Sphaeroma serratum* | BB | 1 | COI |
| Annelida, Polychaeta, Serpulidae | *Spirobranchus triqueter* | BB | 2 | 18S |
| Annelida, Polychaeta, Syllidae | *Syllis gracilis* | BB | 1 | COI |
| Annelida, Polychaeta, Syllidae | *Syllis sp.* | BB | 2 | COI |
| Annelida, Polychaeta, Terebellidae | *Terebella lapidaria* | BB | 1 | 18S |
| Annelida, Polychaeta, Serpulidae | *Vermiliopsis striaticeps* | BB | 1 | 18S |
|  |  |  |  |  |
| Mollusca, Bivalvia, Anomiidae | *Anomia ephippium* | GL | 4 | COI |
| Arthropoda, Decapoda, Carcinidae | *Carcinus aestuarii* | GL | 1 | COI |
| Mollusca, Bivalvia, Cardiidae | *Cerastoderma glaucum* | GL | 1 | COI |
| Mollusca, Gastropoda, Trochidae | *Gibbula adasonii* | GL | 1 | COI |
| Mollusca, Gastropoda, Trochidae | *Gibbula varia* | GL | 5 | COI |
| Annelida, Polychaeta, Nereididae | *Hediste diversicolor* | GL | 26 | COI |
| Annelida, Polychaeta, Nereididae | *Lumbrinereis funchalensis* | GL | 1 | 16S |
| Arthropoda, Crustacea, Majinae | *Maja crispata* | GL | 1 | COI |
| Mollusca, Bivalvia, Pectinidae | *Mimachlamys varia* | GL | 5 | 16S |
| Mollusca, Bivalvia, Mytilidae | *Mytilus galloprovincialis* | GL | 29 | COI |
| Mollusca, Gastropoda, Nassariidae | *Nassarius incrassatus* | GL | 1 | COI |
| Mollusca, Gastropoda, Nassariidae | *Nassarius sp.* | GL | 3 | 16S |
| Mollusca, Gastropoda, Muricidae | *Ocenebra erinaceus* | GL | 1 | COI |
| Mollusca, Gastropoda, Trochidae | *Osilinus turbinatus* | GL | 4 | COI |
| Mollusca, Bivalvia, Ostreidae | *Ostrea edulis* | GL | 4 | COI |
| Arthropoda, Decapoda, Grapsidae | *Pachygrapsus marmoratus* | GL | 1 | COI |
| Arthropoda, Decapoda, Paguridae | *Pagurus cuanensis* | GL | 1 | COI |
| Mollusca, Gastropoda, Patellidae | *Patella caerulea* | GL | 18 | COI |
| Mollusca, Gastropoda, Patellidae | *Patella rustica* | GL | 2 | COI |
| Mollusca, Gastropoda, Patellidae | *Patella ullyssiponensis* | GL | 3 | COI |
| Arthropoda, Cirripedia, Balanidae | *Perforatus perforatus* | GL | 5 | COI |
| Annelida, Polychaeta, Polynoidae | *Polynoe sp.* | GL | 1 | 16S |
| Annelida, Polychaeta, Sabellidae | *Sabella pavonina* | GL | 1 | COI |
